# A Day in the Life of *Microcystis aeruginosa* Strain PCC 7806 as Revealed by a Transcriptomic Analysis

**DOI:** 10.1371/journal.pone.0016208

**Published:** 2011-01-19

**Authors:** Cécile Straub, Philippe Quillardet, Julia Vergalli, Nicole Tandeau de Marsac, Jean-François Humbert

**Affiliations:** 1 Institut Pasteur, Collection des Cyanobactéries, Paris, France; 2 Institut Pasteur, Unité de Dynamique du Génome, Paris, France; 3 Institut Méditerranéen d'Ecologie et de Paléoécologie, UMR-CNRS 6116, Ecologie des Eaux Continentales Méditerranéennes, C31, Université Paul Cézanne, Faculté de St-Jérôme, Marseille, France; 4 Laboratoire de Chimie Bactérienne, CNRS-UPR9043, Université d'Aix-Marseille, Marseille, France; 5 INRA, UMR BIOEMCO, Site de l'ENS, Paris, France; Kyushu Institute of Technology, Japan

## Abstract

The cyanobacterium, *Microcystis aeruginosa*, is able to proliferate in a wide range of freshwater ecosystems and to produce many secondary metabolites that are a threat to human and animal health. The dynamic of this production and more globally the metabolism of this species is still poorly known. A DNA microarray based on the genome of *M. aeruginosa* PCC 7806 was constructed and used to study the dynamics of gene expression in this cyanobacterium during the light/dark cycle, because light is a critical factor for this species, like for other photosynthetic microorganisms. This first application of transcriptomics to a *Microcystis* species has revealed that more than 25% of the genes displayed significant changes in their transcript abundance during the light/dark cycle and in particular during the dark/light transition. The metabolism of *M. aeruginosa* is compartmentalized between the light period, during which carbon uptake, photosynthesis and the reductive pentose phosphate pathway lead to the synthesis of glycogen, and the dark period, during which glycogen degradation, the oxidative pentose phosphate pathway, the TCA branched pathway and ammonium uptake promote amino acid biosynthesis. We also show that the biosynthesis of secondary metabolites, such as microcystins, aeruginosin and cyanopeptolin, occur essentially during the light period, suggesting that these metabolites may interact with the diurnal part of the central metabolism.

## Introduction

*Microcystis aeruginosa* is one of the most common bloom-forming cyanobacteria in freshwater ecosystems, and is widely distributed on the five continents. Members of this genus are known to be able to synthesize numerous secondary metabolites, including microcystins, which are hepatotoxins frequently involved in animal and human poisoning [1], [2]. The genomes of two *M. aeruginosa* strains (PCC 7806 and NIES-843) have recently been sequenced [3], [4], revealing their high plasticity, due in part to the presence of numerous, long, repeated sequences and transposase genes.

The annual life cycle of *M. aeruginosa* is characterized by two distinct phases. The first is a benthic phase, during which it lives on sediment during the Winter and the early Spring, and the second a planktonic phase, during which it lives in the water column during Summer and Autumn [5]. During this second phase, *M. aeruginosa* cells are organized in colonies, which carry out daily vertical migrations in the water column [6]. These vertical migrations are due to hydrodynamic processes, and also to the ability of these microorganisms to regulate their buoyancy [7], [8]. One of the most spectacular effects of this process is the accumulation of *M. aeruginosa* colonies in the top few centimeters of the water column in the early morning, and their subsequent progressive disappearance from the surface over the course of the day. The light exposure of *M. aeruginosa* cells can be very limited due to these vertical migrations and to low light levels in the water column when high cyanobacterial biomass reduces water transparency.

This limited exposure to light can have very important consequences for the metabolism of this photosynthetic microorganism, and may have resulted in adaptative mechanisms to ensure the optimal use of the light available. For example, two recent publications have suggested that *M. aeruginosa* is better able to tolerate lack of light than green algae [9], [10]. However, little firm information is available about the metabolism of this species during the light/dark cycle. Microarray technology, which provides an overall view of gene expression over time, is one of the best ways to obtain such data. Several recent publications on three cyanobacterial species, *Synechocystis* sp. strain PCC 6803 [11], [12], *Cyanothece* sp. strain 51142 [13] and *Prochlorococcus marinus* strain MED4 [14] have demonstrated the effectiveness of this approach.

In the present work we set out to construct a microarray based on the genome of *M. aeruginosa* strain PCC 7806 in order to study the dynamics of gene expression in this cyanobacterium during the light/dark cycle.

## Methods

### Strain and culture conditions

*Microcystis aeruginosa* strain PCC 7806 (hereafter designated *M. aeruginosa*), provided by the Pasteur Culture Collection of Cyanobacteria (Institut Pasteur of Paris), was grown in BG11 medium [15] containing 2 mM of NaNO_3_ and 10 mM of NaHCO_3_. Cultures were placed in an incubator (INFORS AG, Switzerland) continuously sparged with 1% (v/v) CO_2_ in air, at 22°C, shaken (90 rpm), and exposed to 12 h-12 h light-dark cycles. The photosynthetic photon flux density during the light period (50 µmol photons.m^−2^.s^−1^; cool white OSRAM L 18/640) was measured using a LICOR LI-185B quantum/radiometer/photometer equipped with a LICOR LI-193SB spherical sensor.

### Experimental protocol

Two independent experiments, each with three biological replicates, were conducted. in 1L Erlenmeyer flasks. To train the circadian clock before sampling, the cultures were incubated for 10 days under the same experimental conditions. Cell growth was followed by monitoring the OD_750_ using a Uvikon 933 VIS-US spectrophotometer (Kontron Instruments).

Samples were taken in the middle of the exponential-growth phase (OD_750_ 0.6 to 0.8) of the cultures over a period of 24 h (all times are expressed in terms of a 24-h clock): at T0 (00:00, the middle of dark period), T1 (05:30, *i.e.* 30 min before the transition from dark to light), T2 (06:30, *i.e.* 30 min after the transition from dark to light), T3 (07:30, *i.e.* 90 min after the transition from dark to light), T4 (12:00, the middle of the light period), T5 (18:30, *i.e.* 30 min after the transition from light to dark) and finally T6 (24:00, the middle of the dark period) (Supporting Information S1). T6 of this cycle corresponds to T0 of the next 24-h cycle. For each time point, 50 ml of the cultures of each replicate were collected, and centrifuged (15000 *g*, 2 min, 4°C). The cell pellets were then frozen in liquid nitrogen, and stored at −80°C until used for RNA extraction. In the first experiment, samples were collected only at times T0, T2, T4, T5 and T6, whereas in the second experiment all the time points were sampled.

### RNA isolation

Total RNA was isolated and purified using the kit RiboPure™-Bacteria (Ambion) according to the manufacturer's instructions. Cell pellets were resuspended in 750 µl RNAwiz, and 500 µl Zirconia Beads were added. The bacterial cells were lysed for 200 s (10×20 s with 1 min intervals on ice) in a FastPrep FP120 (Qbiogene) at speed 5 m.s^−1^. DNase I treatment was not performed. RNA concentrations were quantified using a NanoDrop ND-1000 Spectrophotometer (NanoDrop Technologies), and the quality of the RNA extracts was checked using the Agilent 2100 Bioanalyzer, RNA 6000 Nano reagents and RNA Nano Chips (Agilent Technologies) according to the Manufacturer's instructions.

### cDNA synthesis and Cy-dye labeling

Five µg of the purified total RNA was used for the random priming of cDNA synthesis and fluorescence labeling (Cy3 or Cy5 dye, Amersham, GE Healthcare) using the SuperScript™ Indirect cDNA Labeling System (Invitrogen) according to the manufacturer's instructions. Indirect labeling relies on the incorporation of aminoallyl-modified nucleotides into cDNA derived from the target RNA. The aminoallyl side chains were then used to label the cDNA with either Cy3 or Cy5. The efficiency of the labeling procedure was analyzed by a NanoDrop ND-1000 spectrophotometer (NanoDrop technologies). The labeled cDNA was used immediately for hybridization to a microarray.

### Construction of *Microcystis aeruginosa* microarrays

The transcriptomic study was performed using an Agilent 4X44K two-color microarray (Agilent technologies). Each slide contained 4 arrays each with 44,000 spots. Specific oligonucleotides (60-mers) were designed for 5085 protein-coding genes corresponding to 96% of the total predicted protein-coding genes in the annotated genome of the *M. aeruginosa* [3]. Oligonucleotide probes for each gene were designed by the eArray protocol (Agilent technologies). Five, four, three, two and one oligonucleotide probes were defined for 68%, 9%, 11%, 9% and 3% of genes, respectively. Each oligonucleotide was randomly printed in duplicate across one array by means of the SurePrint technology (Agilent Technologies).

### Hybridization and scanning of DNA microarrays

Hybridization was performed according to the protocol for the two-color microarray-based gene expression analysis recommended by Agilent Technologies. Briefly, Cy3-labeled cDNAs (150 pmol) from the control culture (T0) was mixed with Cy5-labeled cDNAs (150 pmol) from other time points (T1 to T6), and they were hybridized to one array. The color-swapped cDNA samples (i.e. the same samples with the reverse combination of dye labels) were hybridized to the other array on the same glass slide. The resulting mixture was hybridized to an array at 65°C for 17 h. Following hybridization, each slide was washed as recommended by Agilent. After the slide had been air dried, it was scanned using a GenePix 4200AL scanner (Axon Instruments, Union City, Calif.) at 5 µm resolution. The signal intensity of each spot and its local background were determined using GenePix Pro software (Version 6, Axon Instruments). The net signal intensity was calculated by subtracting the median signal intensity of all the pixels within the local background area from the median signal intensity of all the pixels within the spot area. All the spot areas recognized by the automatic alignment function of the GenePix Pro were confirmed visually. The flagged spots were not used for subsequent data analysis.

### Microarray data analyses

Any bias in signal intensity between the two fluorescent dye channels in a microarray was normalized by locally weighted linear regression analysis or Lowess normalization [16] using MIDAS (freely available from http://www.tm4.org/midas.html). For all normalizations, the smoothing parameter was set to 0.33. Low-intensity spots (Cy3 or Cy5 intensity <2500) were filtered and then, pairs of flip-dye data were subjected to a flip-dye consistency check in which data outside of a two standard-deviations cut-off were removed. The relative expression level of a gene at a given time point was calculated as log2 (Cy5/Cy3), where Cy5 and Cy3 were normalized signal intensities from experimental and control cDNAs.

Several probes (usually, five) were designed for each protein-coding gene, and as two independent experiments were performed in triplicate (biological replicates) and swapping two colors (CY-3/CY-5, CY-5/CY-3, technical replicates), it follows that the transcript abundance obtained for each gene at each sampling time was based on 12 to 60 measurements.

Statistical analyses of the data were performed using the Significance Analysis of Microarrays (SAM) algorithms [17] in MeV (http://www.tm4.org/mev.html), on the median of log2-transformed signal ratios (fold change) of the replicate spots. This analysis was done gene by gene (time point TX/T0 tested against time point T0/T0; One class analysis), but also by taking into account all the genes in the same analysis (Multi class analysis). SAM analysis uses repeated permutations of the data to find out whether the expression of any genes is significantly related to the response. The data for each gene are permuted, and a t-test is computed for both the original and the permuted data for each gene. One valuable feature of SAM is that it gives estimates of the False Discovery Rate (FDR), which is the proportion of genes likely to have been picked out by chance as being significant. Stringent parameters were applied for this criteria because we chose a delta value so that estimated FDR was equal to zero. Finally, a gene was considered as presenting a significant variation in its transcript abundance when it was found significant by SAM analysis (One class or Multi class) for at least one sampling time.

The time point T0 consisted in examining one sample against itself (T0/T0), so that the log2-transformed signal ratio for each gene is expected to be near the value 0. As a control, we have checked using SAM that at time T0, no gene displayed a mean variation of their expression level that was significantly different from 0.

## Results

### General overview

During the normalization step, a strong dye bias (CY-3/CY-5 data very different from CY-5/CY-3 data) was found for the genes displaying very high expression values (Table S1). These values were eliminated from the analysis by applying a flip dye consistency check after the normalization step, a function provided by the MIDAS software. As a result, 70 genes were eliminated from the analysis. For 258 genes, data were only available at certain time sampling points, whereas a complete time series data was obtained for 4757 genes. Very good overall reproducibility of the results was found for these genes, as illustrated for example for *hypD* in Supporting Information S1.

**Figure 1:**
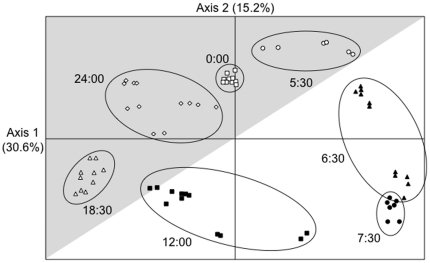
Principal component analysis performed on the transcript abundance values of the 4757 selected genes, estimated in all samples of the two independent experiments. The projection of the samples onto the two most informative axes (Axis 1 and 2) is shown and the inertia value of each axis is provided. At each sampling time, all replicates are represented by the same symbols and grouped in the same ellipse. The gray shaded part of the figure represents the dark period of the light/dark cycle.

To obtain an overview of the global pattern of gene expression during the 24-h light/dark cycle, we performed a principal component analysis (PCA) on the transcript abundance values of the 4757 genes (displaying a complete time series data) obtained for each replicate at each sampling time. The projection of these points onto the plane defined by the first two axes accounting for 45.8% of the total variance (Fig. 1), displayed a spatial ordination of the samples during the light/dark cycle. For each sampling time, all the replicates of the two independent experiments were grouped together and clearly separated from those for the other sampling times. Moreover, the spatial ordination of these points described a clockwise circle beginning at 00:00 and finishing at 24:00.

### Temporal dynamics of the transcriptome

**Figure 2:**
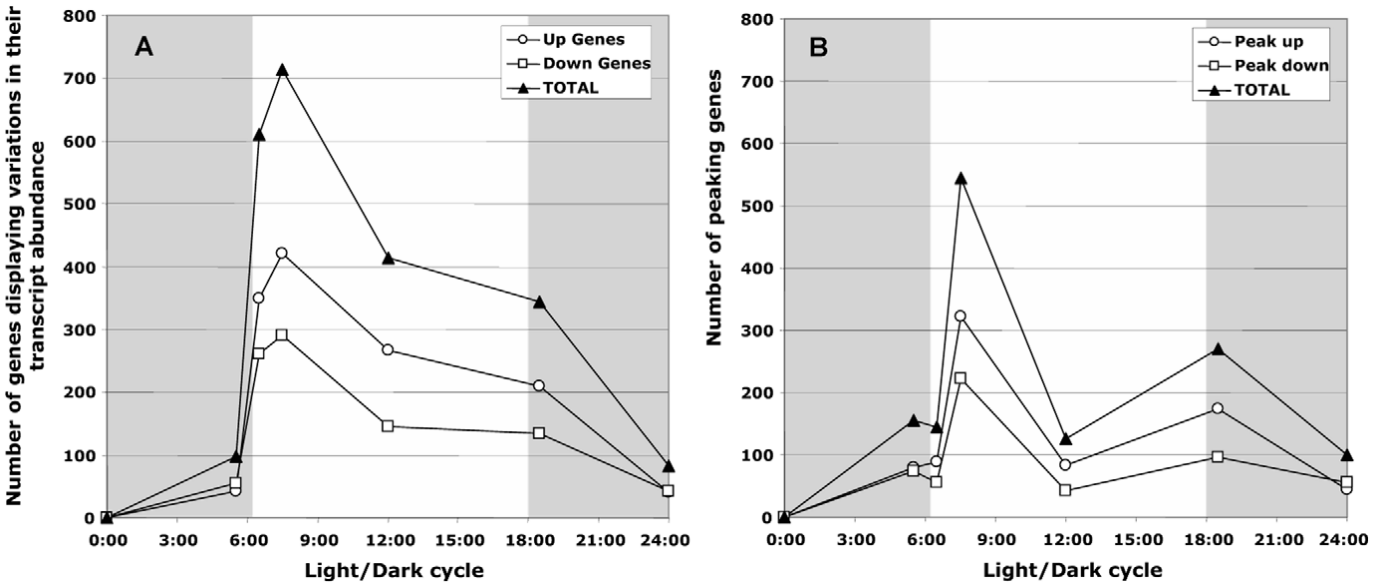
Temporal distribution of genes displaying significant changes in their transcript abundance vs. that at T0. **A.** Number of genes displaying an increase (Up genes) or a decrease (Down genes) in their transcript abundance during the light/dark cycle. **B.** Number of genes displaying a zenith of their transcript abundance (Peak up) or the nadir of their transcript abundance (Peak down) during the light/dark cycle. The gray shaded part of the figure represents the dark period of the light/dark cycle.

From the statistical analyses, it appeared that the transcript levels of 1344 genes varied significantly at least at one sampling time during the light/dark cycle, which is equivalent to 25% of the *M. aeruginosa* protein-coding genes identified in the genome. The complete data set is provided in Table S2. We found also that among these 1344 genes, 40% displayed a zenith (peak-up) or a nadir (peak down) in their transcript abundance at 07:30 (*i.e.* 1h30 after the transition from dark to light) knowing that most of them were characterized by a maximum transcript abundance (Fig. 2A). For most of these genes, changes could already be observed at 06:30, 30 min after the lights had been switched on. At 12:00, the number of genes differentially expressed steadily decreased, and then remained stable untill 18:30 (i.e. 30 min after the transition from light to dark). In the middle of the night (24:00), around 100 genes still displayed significant variations in their expression relative to T0. Overall, the maximum or minimum values of the expression of all the genes usually occurred at 07:30, with a second peak at 18:30 (Fig. 2B). This indicates that light/dark (or dark/light) transition triggers major changes in the expression of the *M. aeruginosa* genome.

### Transcriptome analysis and clusters of orthologous genes (COG) categories

**Figure 3:**
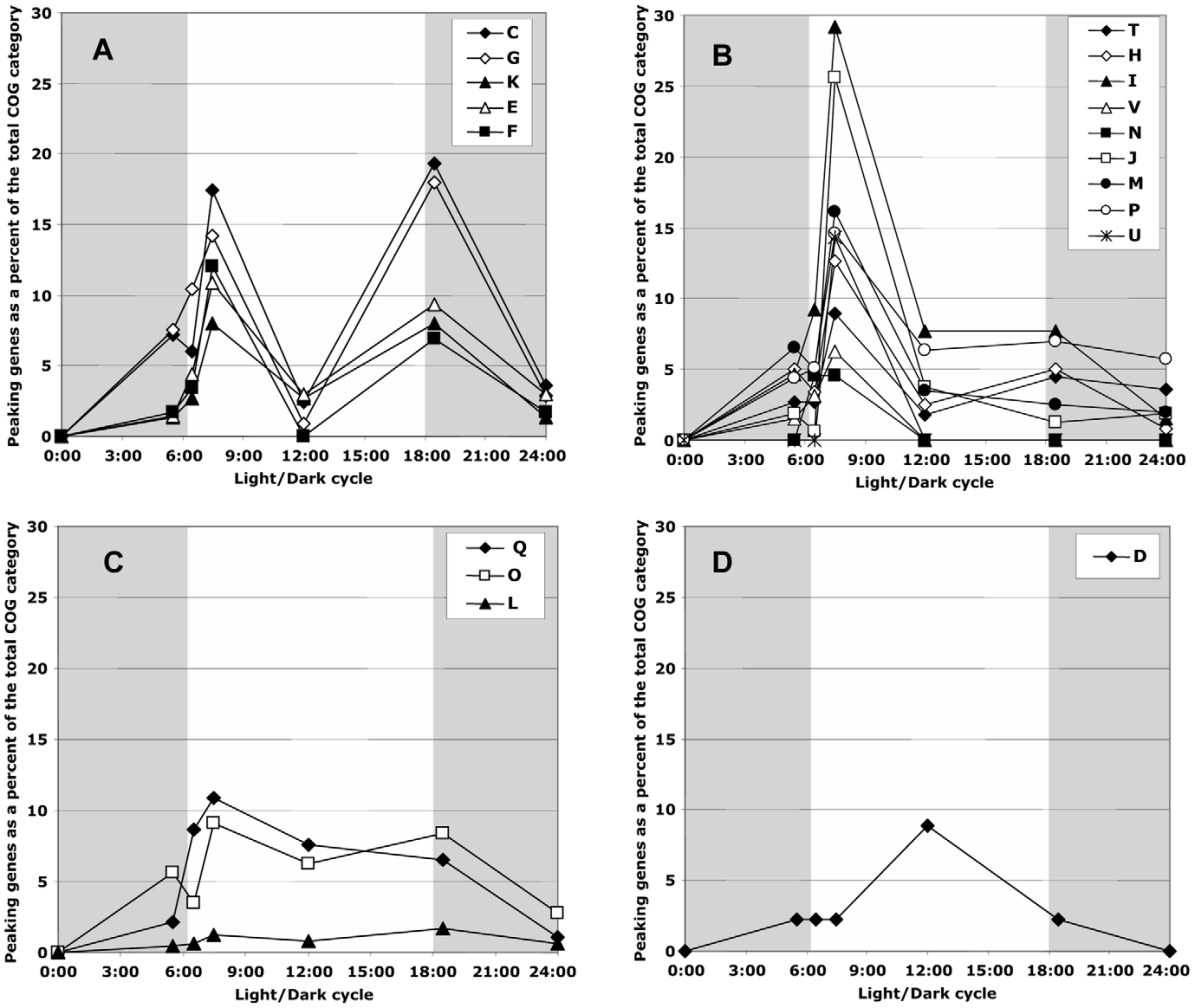
Different patterns in peaking genes classified with regard to their COG functional category (C, D, E, F. G, H, I, J., K, L, M, N, O, P, Q, T, U, V). **A.** Genes peaking both at 7:30 and 18:30. **B.** Genes only peaking at 7:30. **C.** Genes showing maximum expression throughout the light period. **D.** Gene only peaking at12:00. For each COG (cluster of orthologous genes) category, we estimated the percentage of genes (belonging to this category) displaying a zenith (Peak up) or a nadir of their transcript abundance (Peak down) during the light/dark cycle. The gray shaded part of the figure represents the dark period of the light/dark cycle.

The 1344 genes displaying significant variations during the light/dark cycle were further sorted using COG (cluster of orthologous genes) categories [18]. It was possible to assign a COG category to 853 (63%) of these 1344 genes. On the basis of their pattern of expression during the light/dark cycle, the COG-assigned genes could be divided into 4 groups (Fig. 3).

The first group (29% of the COG-assigned genes) contained genes that peaked at both 07:30 and 18:30 (Fig. 3A). These genes belonged to five COG categories (C: Energy production and conversion; E: Amino acid transport and metabolism; F: Nucleotide transport and metabolism; G: Carbohydrate transport and metabolism and K: Transcription), and among them, the highest transcript levels were obtained for genes involved in photosynthesis and energy metabolism.

The second group (36% of the COG-assigned genes) contained genes that peaked only at 07:30 (Fig. 3B). Genes in this group belonged to nine COG categories (H: Coenzyme metabolism; I: Lipid metabolism; J: Translation, ribosomal structure and biogenesis; M: Cell envelope biogenesis, outer membrane; N: Cell motility and secretion; P: Inorganic ion transport and metabolism; T: Signal transduction mechanisms; U: Intracellular trafficking, secretion, and vesicular transport and V: Defense mechanisms;) and among them, the highest transcript levels were found for genes involved in lipid metabolism and translation processes.

The other two groups included genes representing only a small number of COG categories. The third group (10% of the COG-assigned genes) contained genes displaying high transcript levels throughout the light period (Fig. 3C). These genes belonged to three COG categories (L: DNA replication, recombination and repair; O: Posttranslational modification, protein turnover, chaperones and Q: Secondary metabolites biosynthesis, transport and catabolism). The fourth group contained genes belonging to only one COG category (D: Cell division and chromosome partitioning), and displaying high transcript levels in the middle of the light period (Fig. 3D).

Finally, 25% of the COG-assigned genes belonged to categories R and S, which are poorly characterized.

### Carbon transport and energy metabolism

**Figure 4:**
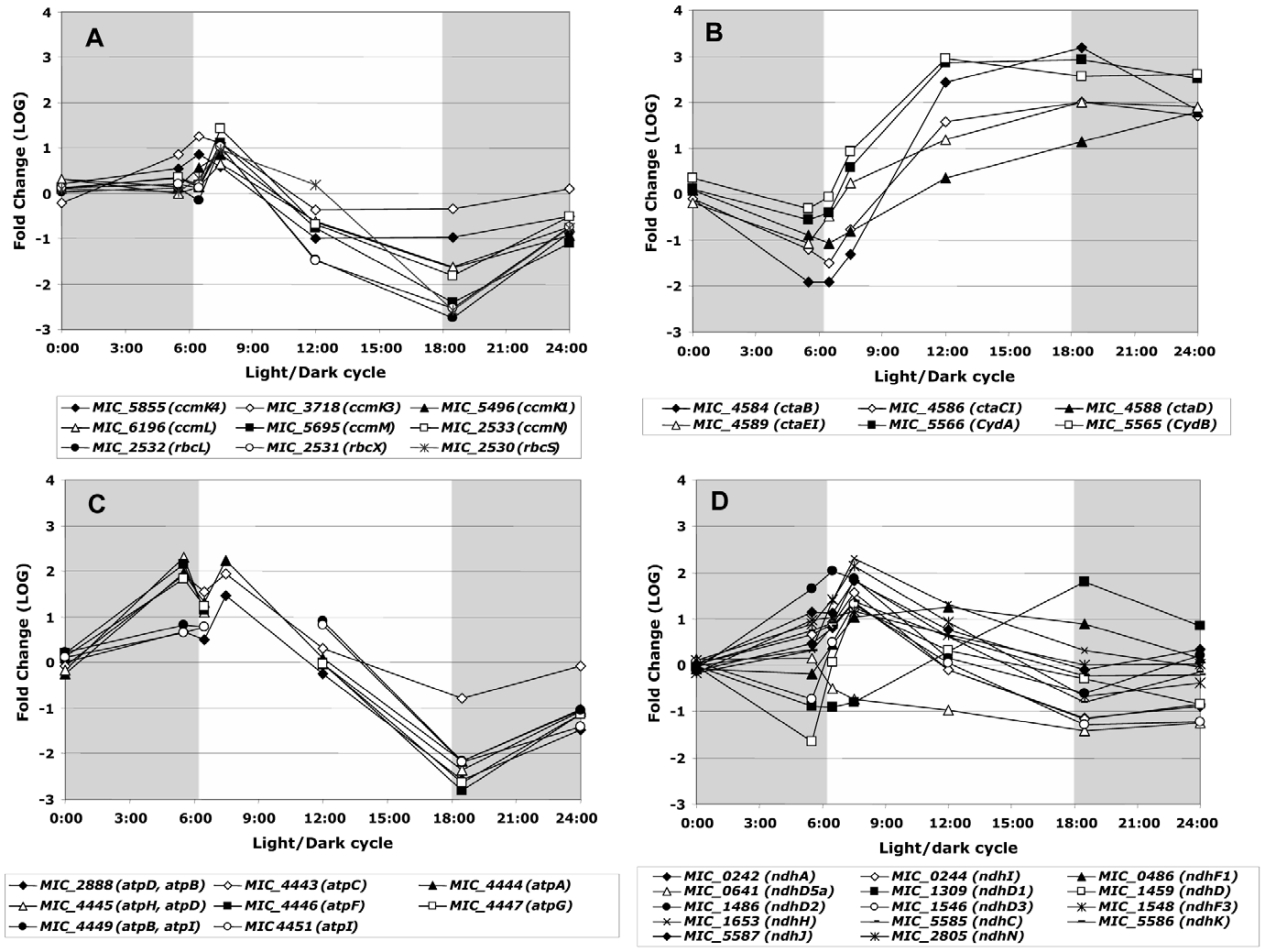
Significant variations in the transcript abundance of genes involved in carbon transport and energy metabolism during the light/dark cycle. **A.** Carbon dioxide concentrating mechanism genes (*ccm* genes) and ribulose bisphosphate carboxylase genes (*rbc* genes). **B.** Respiratory terminal oxidases, cytochrome c oxidases and protoheme IX farnesyltransferase genes (*cta* and *cyd* genes). **C.** ATP synthase genes (*atp* genes). **D.** NADH dehydrogenase genes (*ndh* genes). The gray shaded part of the figure represents the dark period of the light/dark cycle.

Most of the genes with expression peaks at both 07:30 and 18:30 are involved in carbon transport and energy metabolism processes (COG categories C and G). A more detailed analysis shows that transcript abundance of the *ccm* (carbon dioxide concentrating mechanism) and *rbc* (ribulose bisphosphate carboxylase) genes peaked soon after the transition from dark to light and then rapidly decreased after the transition from light to dark (Fig. 4A). Similar patterns of expression were found for most of the genes encoding the *ndh* (NADH dehydrogenase subunits) and for the *atp* genes (ATP synthase subunits) (Figs. 4C and 4D). On the other hand, the opposite pattern of expression was found for genes involved in the respiratory terminal oxidase system (*cta* genes), such as the genes encoding three cytochrome c oxidase subunits and the protoheme IX farnesyltransferase (Fig. 4B).

Only four *pet* genes (cytochrome b_6_f complex) displayed significant variations in their transcript abundance. For the *petCA* gene cluster, the maximum transcript level occurred at 07:30 (Supporting Information S1), while the *petBD* gene cluster peaked at 12:30 and 18:30 (Supporting Information S1). Like *petBD*, but unlike *petAC*, *cydA* and *cydB* (cytochrome *bd*-I quinol oxidase) displayed their lowest transcript levels in the morning, and their highest ones in the afternoon (Fig. 4B). Finally, the *hoxEFUYH* (bidirectional hydrogenase) and the *hypCD* genes (maturation of the enzyme) displayed similar patterns of expression, with transcript levels peaking at the beginning of the dark period (Table S2).

**Figure 5:**
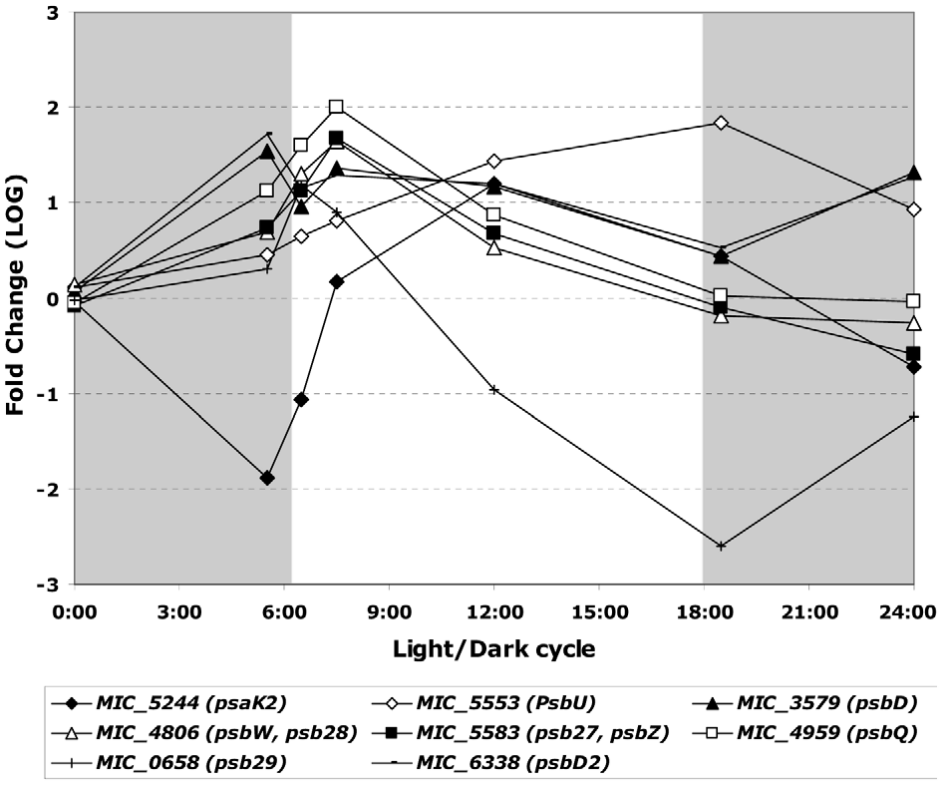
Significant variations in the transcript abundance of genes involved in photosystem I (*psa* genes) and photosystem II (*psb* genes) during the light/dark cycle. The gray shaded part of the figure represents the dark period of the light/dark cycle.

Eleven photosystem I genes (*psa*) and 20 photosystem II genes (*psb*) have been identified in *M. aeruginosa*. These include *psbD*, *psbD2*, *psbQ*, *psbW*, *psb27* and *psb29*, which displayed significant changes in their transcript patterns with maximum values at 06:30 or 07:30 (Fig. 5). On the other hand, *psaK2* transcript abundance was very low at 05:30 and 06:30, but peaked at 12:00. The *psbU* gene displayed a single transcript abundance peak, which occurred at the beginning of the dark period (18:30).

**Figure 6:**
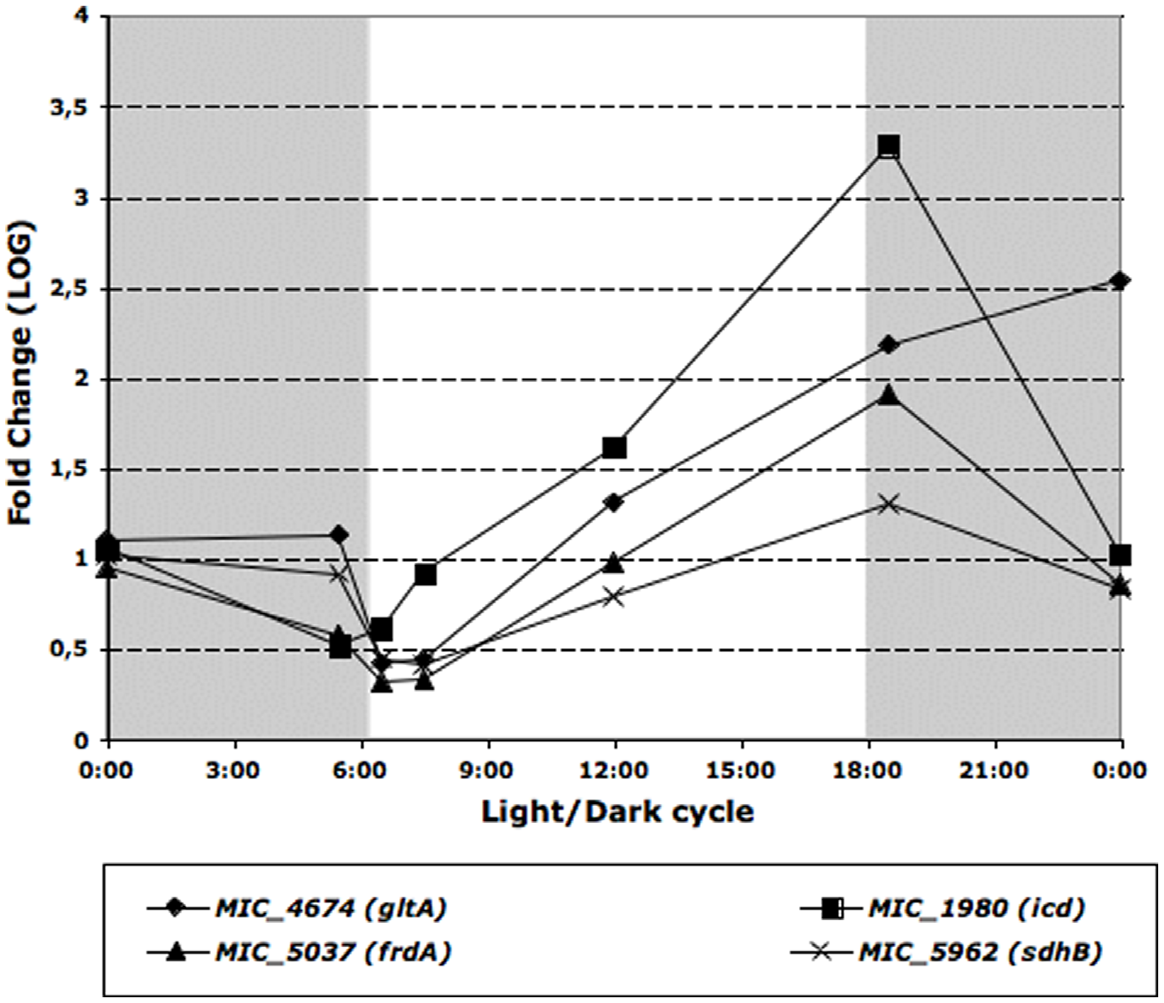
Significant variations in the transcript abundance of genes involved in the TCA pathway during the light/dark cycle. The gray shaded part of the figure represents the dark period of the light/dark cycle.

Finally, when considering the TCA cycle, four genes (isocitrate dehydrogenase(*icd*), citrate synthase (*gltA*), succinate dehydrogenase flavoprotein subunit (*sdhA*) and succinate dehydrogenase iron-sulfur protein subunit (*sdhB*)) among the seven genes described in the *M. aeruginosa* genome for this pathway, displayed significant changes in their transcript abundance, with maximum transcript abundance values during the night and minimum values just at the beginning of the light period (Fig. 6).

### Nitrogen and phosphate transport and metabolism

**Figure 7:**
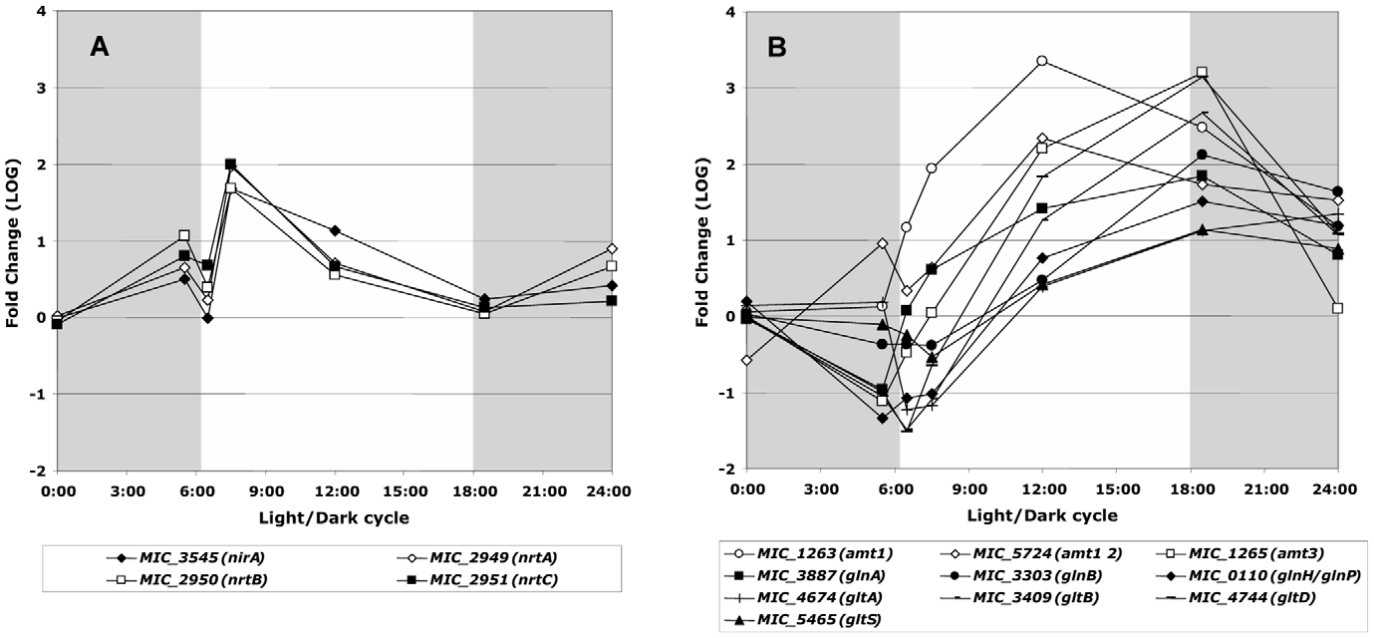
Significant variations in the transcript abundance of genes involved in nitrogen transport and metabolism during the light/dark cycle. **A.** Nitrite reductase (*nirA*) and the nitrate/nitrite transport (*nrt*) genes. **B.** Ammonium permease (*amt*), transport and synthesis of glutamate (*glt*) and glutamine (*gln*) genes. The gray shaded part of the figure represents the dark period of the light/dark cycle.

The transportation and metabolism of nitrate and phosphate are very important for freshwater cyanobacteria as these nutrients are generally growth limiting in continental aquatic ecosystems. While the transcript levels of *narB* (nitrate reductase) did not significantly change during the light-dark cycle, the expression of the *nirA* (nitrite reductase) and *nrt* (nitrate/nitrite transport) genes both peaked soon after the light was switched on (Fig. 7A). In contrast, the expression of the three ammonium permease genes occurred later (*amt1* and *amt2*: max value at 12:00; *amt3*: max value at the beginning of the dark period) (Fig. 7B). The same pattern of expression as for *amt3* was found for the genes involved in the transport and synthesis of glutamate (*gltS* and *gltBD*, respectively) or of glutamine (*glnH* and *glnA*, respectively), as well as for the *gltA* (citrate synthase) and *icd* (isocitrate dehydrogenase), two genes required for the synthesis of 2-oxoglutarate, and for the *glnB* gene that codes for the signal transduction PII protein, which is known to coordinate nitrogen and carbon metabolism (Fig. 7B and Table S2).

It is worth noticing that *gltBD* (NADH-dependent glutamate synthase subunits) and *glsF* (ferredoxin-dependent glutamate synthase) display opposite patterns of expression, with a high level of *glsF* transcripts observed from 06:30 to 12:00 followed by a decrease during the dark period, which is when the *gltBD* transcripts reached their maximum value. Like the *nirA* and *nrt* genes, the transcript levels of *argJ* (a bifunctional enzyme involved in the first step of the arginine biosynthetic pathway from glutamate) display a sharp increase followed by a decrease during the light-dark cycle, peaking at 07:30 (Fig. 7B and Table S2).

Three ABC-type phosphate transporter genes (*pst*) displayed only slight variations in their transcript abundance, which was characterized by a slight increase between 00:00 and 07:30, followed by a decrease. In contrast, the two phosphate regulator genes *phoB* (a two-component system response regulator of a phosphate sensing system) and *phoU* (a phosphate uptake regulator) displayed contrasting patterns of expression, with a marked decrease in *phoB* transcript levels at 12:00 and 18:30, whereas those of *phoU* increased during the night (18:30 and 24:00) (Table S2).

### Cell division and chromosome partitioning

The transcript levels of most COG category D genes (D: Cell division and chromosome partitioning) were high in the middle of the light period. However, their pattern of expression was essentially due to variations in the transcript levels of genes either with unknown functions or only indirectly related to cell division. The transcript abundance of only one gene that may be involved in chromosome partitioning (MIC_1479) peaked in the middle of the light period (Table S2). The transcript abundance of *ftsH*, which encodes a protein essential for cell division and classified as COG category O, peaked at 5:30, just before the dark/light transition, whereas *ftsK/spoIIIE*, which encodes a putative cell division protein, and *minD* which encodes a septum site-determining protein peaked at 6:30, just after the dark/light transition. Similarly, genes encoding rod shape-determining proteins *(mreB*; *mreC*, belonging to the COG category M) displayed a transcript abundance peak at 07:30 (Table S2).

### Secondary metabolites

**Figure 8:**
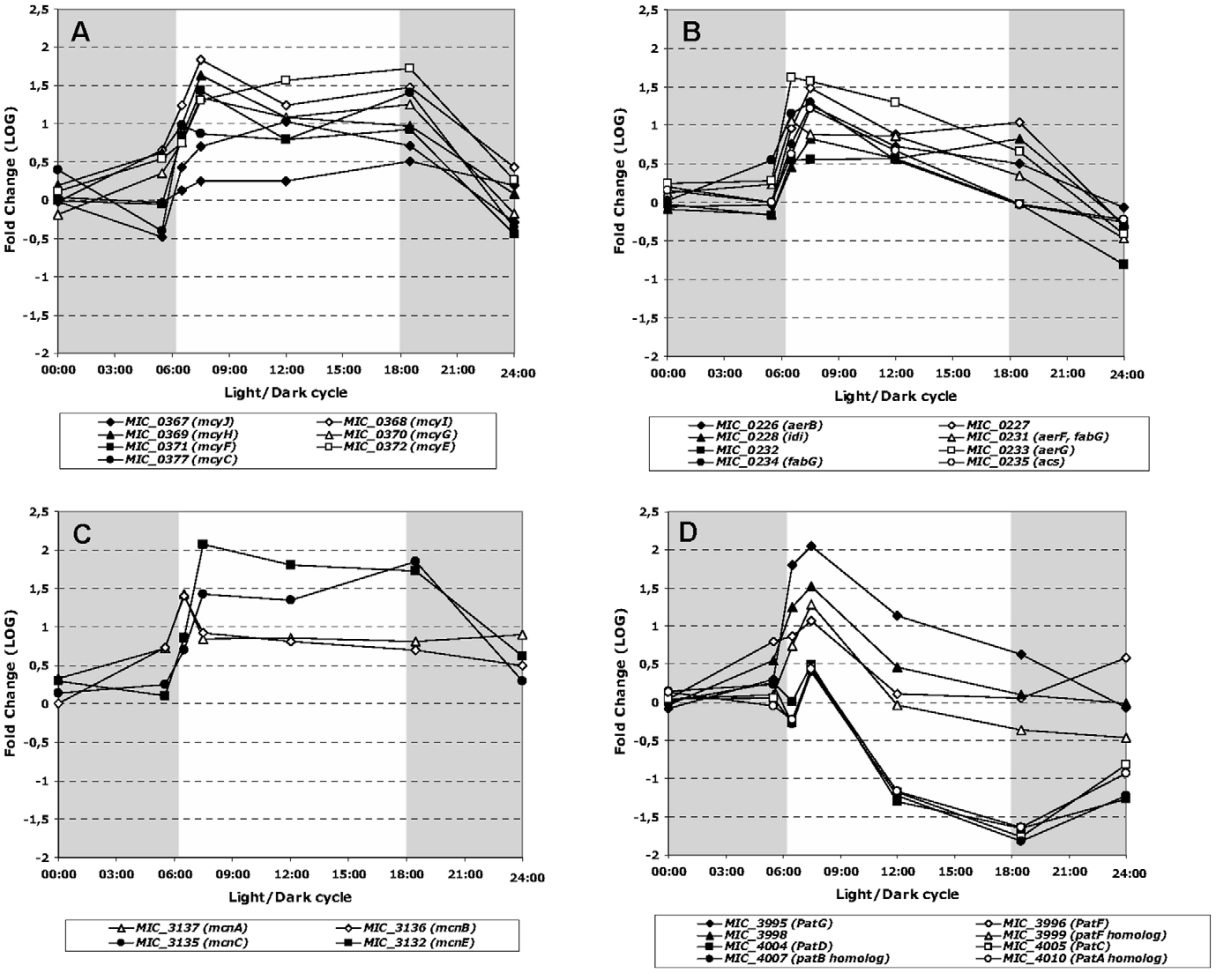
Significant variations in the transcript abundance of genes involved in the biosynthesis of secondary metabolites during the light/dark cycle. **A.** Microcystin gene cluster. **B.** Aeruginosin gene cluster. **C.** Patellamide-like microcyclamide cluster. **D.** Cyanopeptolin gene cluster. The gray shaded part of the figure represents the dark period of the light/dark cycle.

COG analysis of the genes belonging to category Q (Q: Secondary metabolites biosynthesis, transport and catabolism) has shown that most of the genes involved in the biosynthesis of secondary metabolites displayed high transcript levels throughout the light period. More detailed analysis showed that all the genes involved in the biosynthesis of microcystins (*mcy* genes), with the exception of *mcyB* and *mcyD*, were characterized by an increase in their transcript abundance shortly after the light was switched on (at 06:30 and 07:30, respectively), then remained stable or declined slightly until night began (18:30), and finally displayed a sharp decrease when the light was turned off (Fig. 8A). The *mcyB* and *mcyD* genes displayed the same overall transcription patterns, but the changes were not statistically significant due to wide variations in the values obtained for the different replicates. The transcription patterns of the genes involved in the biosynthesis of both aeruginosin and cyanopeptolin were generally similar to those of the microcystin genes, with only a slight difference for the *mcnA* and *mcnB* genes, which displayed a single transcript abundance peak just after the transition from dark to light (06:30), followed by a decrease (Fig. 8B and 8C). Similarly, the transcript levels of the genes required for the biosynthesis of the patellamide-like microcyclamide, also rose just after the transition from dark to light (06:30 and 07:30), and then fell during the light period (Fig. 8D). Finally, the two PKS I gene clusters (PKSI modular/PKSIII and PKSI iterative) did not show any significant variation in their transcript levels during the light/dark cycle (Table S2).

### Regulation of the light/dark cycle (Circadian clock and sigma factors)

**Figure 9:**
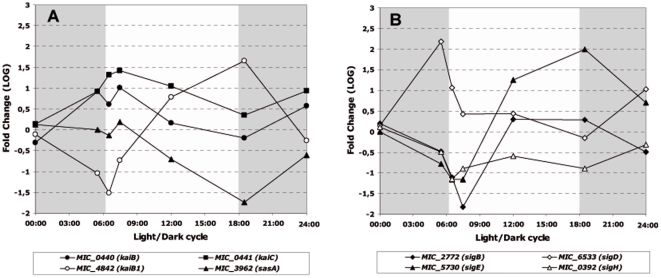
Significant variations in the transcript abundance of genes involved in the circadian clock and sigma factors during the light/dark cycle. **A.** Circadian clock genes (*kai* and *sas* genes). **B.** Sigma factors. The gray shaded part of the figure represents the dark period of the light/dark cycle.

The day/night variations in the abundance of transcripts of numerous genes suggest that they are influenced by both light and an endogenous circadian rhythm. This prompted us to look at the dynamics of the genes involved in the circadian clock (*kai* genes). Whereas the transcript levels of the *kaiA* gene showed no significant modifications during the light/dark cycle, the transcription patterns of the *kaiB* and *kaiC* genes and of the two-component sensor histidine kinase *sasA* gene (KaiC-interacting protein), were very similar (Fig. 9A). In contrast, the *rpaA* gene, which together with *sasA* forms a two-component regulatory system involved in circadian clock regulation, did not display any significant changes in transcript abundance (Table S2). Finally, *kaiB1*, a paralog of the *kaiB* gene, displayed an inverse pattern of expression to *kaiB* and *kaiC* (Fig. 9A), with a low value at the onset of the light period and a high value at the onset of the dark period.

Sigma factors could also significantly influence the light/dark expression of numerous cyanobacterial genes. Among the *sig* genes identified in the genome of *M. aeruginosa* PCC 7806, four sigma factors, *sigB*, *sigD*, *sigE* and *sigH*, displayed significant changes in their transcript levels during the light/dark cycle (Fig. 9B). Very similar patterns of transcript abundance were observed for *sigB* and *sigE*, with minimum values at 07:30 and maximum values at 18:30. The transcript profiles for *sigD* and *sigH* differed both from each other, and from those of *sigB* and *sigE* (Fig. 9B).

## Discussion

As far as we are aware, this study is the first to provide data about the overall expression of the *M. aeruginosa* genome during a light/dark cycle. More than 25% of the genes displayed significant variations in their transcript abundance during the 24 h cycle, most of which occurred following the dark/light transition. Similar results were reported by Stöckel *et al.* [13], who found that nearly 30% of the genes of *Cyanothece sp.* strain 51142 exhibited cycling behavior, and that most of them were involved in core metabolic processes (photosynthesis, respiration, energy metabolism…). On the other hand, for *Prochlorococcus marinus* strain MED4, which has a much smaller genome (≈1700 kb) than either *Cyanothece* or *Microcystis* (≈5000 kb), Zinser *et al*. [14] estimated that 80% of the genes displayed cyclic expression during the diel cycle. The core genome of *Prochlorococcus* has been estimated to consist of >1200 genes [19], which corresponds to more than 70% of the MED4 genome, which means that most of the genes expressing cyclic variations in MED4 belong to the core genome. Similarly, only 176 of the 1344 genes displaying significant variations in the study reported here were specific to *Microcystis* (i.e. were not found in any of the other twelve cyanobacterial genomes in the study of Frangeul *et al*. [3]). Collectively, these findings indicate that in cyanobacteria most of the genes displaying cyclic variations during a dark/light cycle belong to the core genome, and so are involved in basic metabolic processes. This also suggests that in cyanobacterial genera with large genomes, such as *Microcystis* or *Cyanothece*, numerous genes are likely to be involved in light-independent adaptation processes. However, as found in *Cyanothece* [13], [20], our findings showed that light/dark transitions play an important role in the metabolism of *Microcystis*.

Cyanobacteria are known to be able to perform oxygenic photosynthesis and respiration simultaneously [21], but a contrast between night and day was obvious in the transcript abundance of the genes involved in photosynthesis and of those involved in respiration. Indeed, the *ccm* and *rbc* genes (oxygenic photosynthesis) displayed maximum transcript abundance after the switch to light (06:30 and 07:30), whereas the *cta* genes (respiration), displayed maximum transcript abundances when the dark period began (18:30). Similar results were found for *rbcL* in *Prochlorococcus* [14] and *Cyanothece* [20] during a light-dark cycle. In the same way, most of the genes coding for components of oxidative phosphorylation, such as NADH dehydrogenase (*ndh* genes) and ATP synthase (*atp* genes), were up-regulated at the beginning of the light period, as reported in *Synechocystis* sp. strain PCC 6803 [12], [22]. In cyanobacteria, ATP and NADPH are used in many metabolic processes including CO_2_ fixation. In particular, NADPH appears to be used preferentially for carbon fixation processes, rather than for respiration [21], [23]. On the other hand in cyanobacterial thylakoids, the main respiratory electron transport activity into the plastoquinone pool involves the succinate dehydrogenase genes (*sdh*) [23]. These genes, and others from the TCA branched pathway, displayed the same pattern of transcription as those involved in respiration (data not shown). Finally, the responses of bidirectional hydrogenase genes (*HoxFUYH* and *hypCD*) support the hypothesis that these enzymes may be involved in fermentation or respiration, rather than in photosynthesis (see the review of Tamagnini *et al*. [24]).

With regard to nutrient uptake, if, as expected, carbon metabolism occurs at the beginning of the light period, this makes it even more surprising to find ammonium assimilation occurring at the end of the light period and during the first few hours of the night. Ammonium assimilation has been clearly shown to occur in cyanobacteria (*e.g.* [25], [26]). In *Spirulina platensis*, Boussiba [27] found that ammonium uptake proceeded at the same rate in light and in darkness. In contrast, our result suggests that the assimilation of carbon and of ammonium occur at different times, like CO_2_ and N_2_ fixation in *Cyanothece* sp. ATCC 51142 [13] and in *Trichodesmium* sp. strain IMS-101 [28]. The preferential assimilation of ammonium during the night could be explained by the ecological conditions that drive the cell biology of *Microcystis*. This species is known to perform vertical migrations in the water column, and to be located lower down in the water column during the first few hours of the dark period [6]. However, while an increase of phosphorus concentrations with depth has been well documented for many lakes, nothing suggests that ammonium concentrations display similar changes.

Altogether, these data suggest that light is not the sole trigger for the transcription of genes involved in photosynthesis and respiration. Indeed their transcription may also be under the control of an endogenous circadian clock, which makes it possible to anticipate their regulation in order to ensure an optimum efficiency of the processes when the changes in environmental conditions arise. The circadian clock is an endogenous biological timing mechanism found in almost all organisms [29], however Cyanobacteria are the simplest organisms known to exhibit circadian rhythms. The *kaiABC* genes have been identified as essential genetic components for circadian oscillation in most cyanobacteria [30]. We did indeed find oscillations in the transcript abundance of the *kaiB* and *kaiC* genes, whereas there was no such significant variation for the *kaiA* gene, a regulator of *kaiBC* transcription [31], during the light/dark cycle. This is consistent with previous findings showing that the transcript levels of *kaiBC* displayed high amplitude oscillations, whereas the oscillations of the *kaiA* mRNA were characterized by a lower amplitude [31]. In addition to the *kaiABC* locus, *M. aeruginosa* also possesses two paralogs of *kaiB*, and one of *kaiC* located at other loci. One of the *kaiB* paralogs exhibited strong periodicity, with an inverse pattern to that of the *kaiABC* locus, but it is not known to what extent this *kaiB* paralog is involved in the circadian clock process. These data do not allow us to pinpoint which specific genes of *M. aeruginosa* are light induced or circadian controled. A recent paper [32] on *Cyanothece* suggests on the basis of new analytical methods that most of the genes displaying peak expression in the middle of the light period are light responsive, whereas those that are up-regulated at the onset of the dark period are circadian controled. Whether this holds true for *M. aeruginosa* or not remains to be experimentally demonstrated.

With regard to the sigma factors, we found interesting conflicting results in the transcript abundances of the *sigD* and *sigE* genes. As reported by Imamura and Asayama [33], in cyanobacteria *sigD* contributes to the light-induced transcription of the *psbA2* and *psbA3* genes. This is consistent with the finding that *psbA2* transcript abundance peaks at 05:30. More generally, the transcription pattern of the *sigD* gene, which peaks at 05:30, seems to suggest that the SigD function could be crucial for all genes displaying a light-induced transcription in cyanobacteria [33]. With regard to the *sigB* and *sigE* genes, the fact that the transcripts peak at 18:30 is consistent with the putative function of these sigma factors in the regulation of nitrogen metabolism (Imamura & Asayama, 2009). Indeed, as we show in the present study, many genes involved in nitrogen metabolism (*amt3*, *glnA*, *gltS…*) display similar transcript abundance patterns to the *sigB* and *sigE* genes. However, our findings on the transcription of *mcy* genes and of some other genes involved in the production of secondary metabolites, do not confirm the hypothesis that SigD and SigE are involved in regulating these NRPS genes.

From a more general standpoint, this first transcriptomic study of a *Microcystis* species has shown that the metabolism of this cyanobacteria is clearly compartmented between the light period, during which carbon uptake, photosynthesis, and the reductive pentose phosphate pathway permit the synthesis of glycogen, and the dark period during which glycogen degradation, the oxidative pentose phosphate pathway, the TCA cycle and ammonium uptake permit amino acid biosynthesis. This organization of the central metabolic pathways is very similar to that described in *Cyanothece* sp. strain ATCC 51142 [13], a unicellular marine diazotrophic species, suggesting that many cyanobacterial species may share the same central metabolic organization. More specifically, *Microcystis* is able to produce secondary metabolites, particularly microcystins. The fact that all the genes involved in the biosynthesis of microcystin, and also that of aeruginosin and to some extent that of cyanopeptolin, displayed the same expression pattern with clear upregulation during the light period suggests that these metabolites are essentially produced during the light period and that they may interact with the diurnal part of the central metabolism.

Another question to address is the representativeness of all the results presented in this study compared to the conditions encountered by the *Microcystis* cells in natural environments. Even if we have chosen to apply a moderate light irradiance to the cultures, to reproduce at the best the natural irradiance conditions, two main differences persist. The first one is the absence of progressive increase and decrease in the light intensity during the transition from dark to light and light to dark, respectively. Such experimental conditions could provoke a stress and explain that for several genes (*atpA* and *atpG*, for example) a decrease in transcript abundance was temporarily observed at 06:30. Another difference with natural conditions concerns the continuous exposition of the cells to light for 12 hours, whereas in natural aquatic ecosystems, *Microcystis* cells are only located at the top of the water column during the first hours of the day, before to be mixed in this water column and to have a discontinuous access to the light during the rest of the day. Interestingly, in spite of these limitations, our study clearly shows that a large part of the carbon and energy metabolism is performed in the first hours of the light period, suggesting the existence of an adaptative process controlling the daily cycle of *M. aeruginosa* in natural ecosystems.

## Supporting Information

Table S1 **List of genes with missing data.** The second column, gene or gene info, shows either the gene name or brief information about the gene, and the fourth column gives the number of missing data (i.e. the value is one when data are missing data at one sampling time; this value is two when data are missing at two sampling times…). Except for the 70 genes, which have been eliminated by the flip dye consistency check, all the other genes were taken into account in the analyses.
(XLS)

Table S2 **Complete data set of our transcriptomic results.** The second column, gene or gene info, shows either the gene name or brief information about the gene. The fold change (Tx/T0) is given for each gene at each sampling time. The last column (STAT) gives the result of the statistical analysis (sig: significant variation of the gene transcript abundance; ns: non significant variation of the gene transcript abundance).
(XLS)

Supporting Information S1 **1. Design of our experiment.** The figure shows the various sampling times during the light (yellow)/dark (gray) cycle. T1: 0:00; T2: 5:30; T3: 6:30; T4: 7:30; T5: 12:00; T6: 18:00; T7: 24:00. **2. Variations in the transcript abundance values obtained for one gene (*hypD*) during the light/dark cycle.** For the six biological replicates (R1 to R6), all the fold change values obtained at each sampling time for the five oligonucleotide probes designed in this gene were plotted in the figure. The gray shaded part of the figure represents the dark period of the light/dark cycle. **3. Significant variations in the transcript abundance of genes encoding cytochrome b_6_f complex (*pet* genes) during the light/dark cycle.** A: transcript abundance of the *petBD* gene cluster and B: transcript abundance of the *petCA* gene cluster. The gray shaded part of the figure represents the dark period of the light/dark cycle.
(PDF)
