# Supplementary figures and images for: A Day in the Life of Microcystis aeruginosa Strain PCC 7806 as Revealed by a Transcriptomic Analysis
Source: PLoS One. 2011 Jan 19;6(1):e16208. doi: 10.1371/journal.pone.0016208 (PMC3023806; doi:10.1371/journal.pone.0016208)

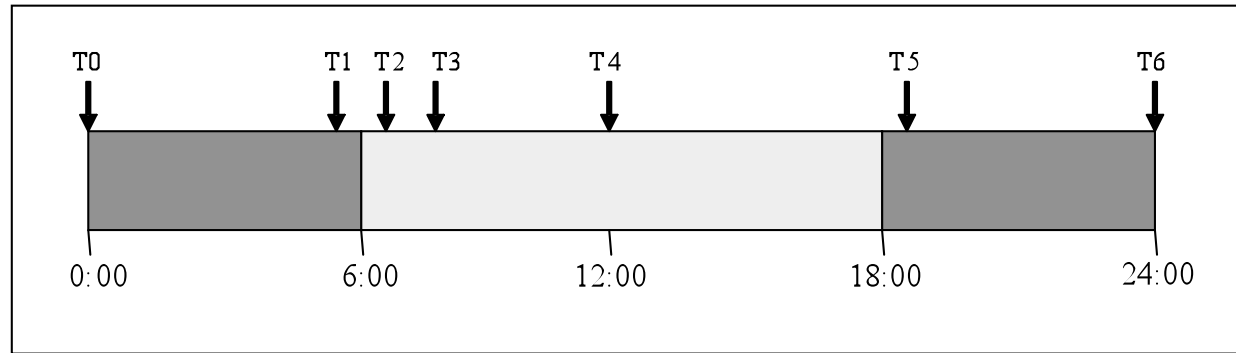

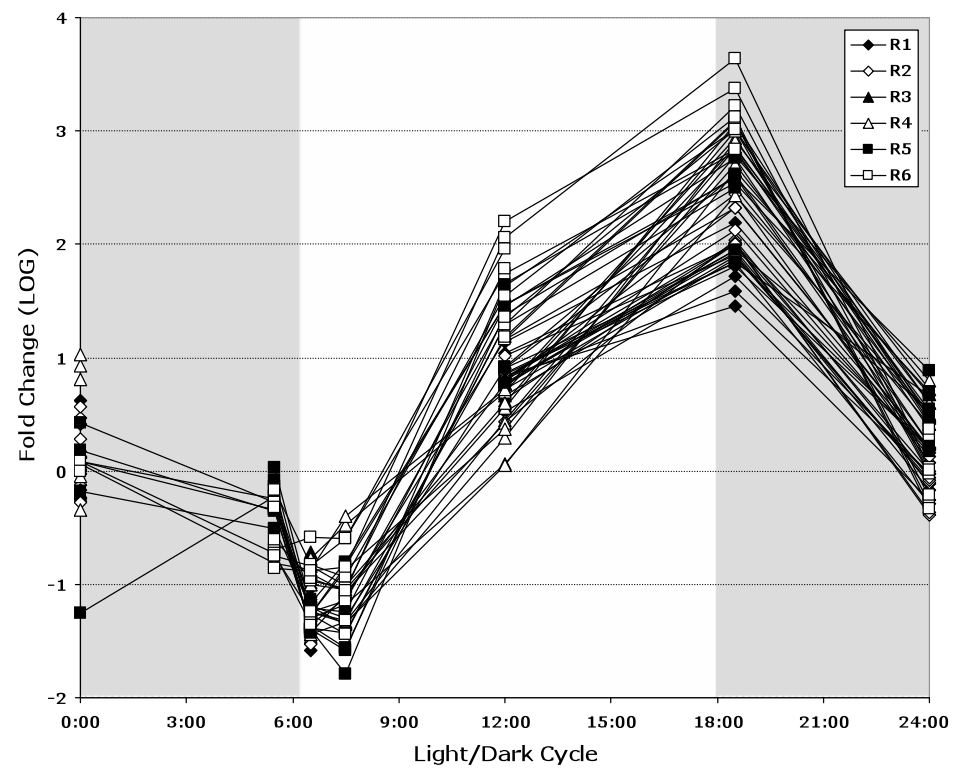

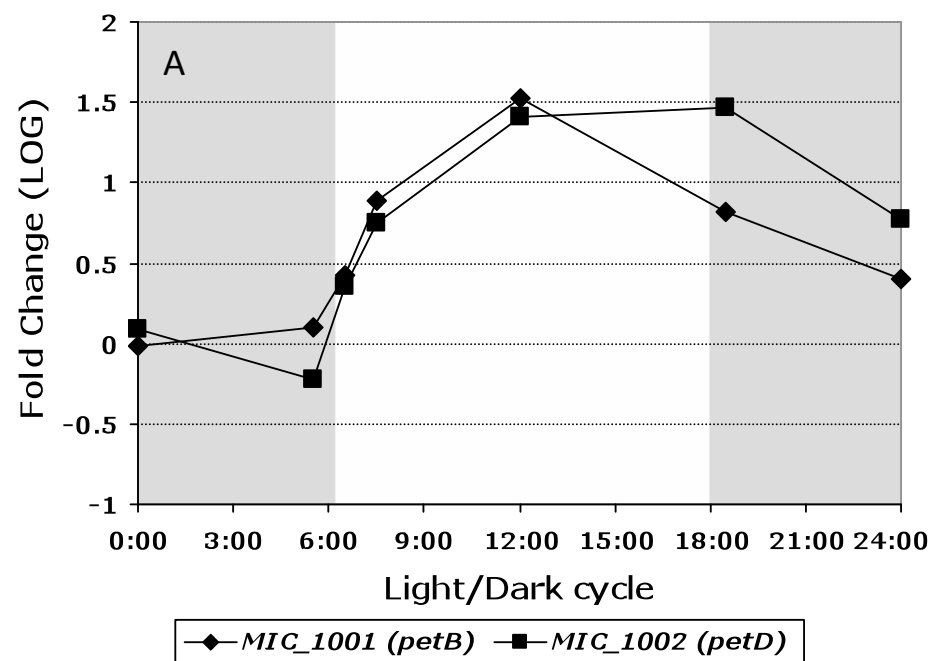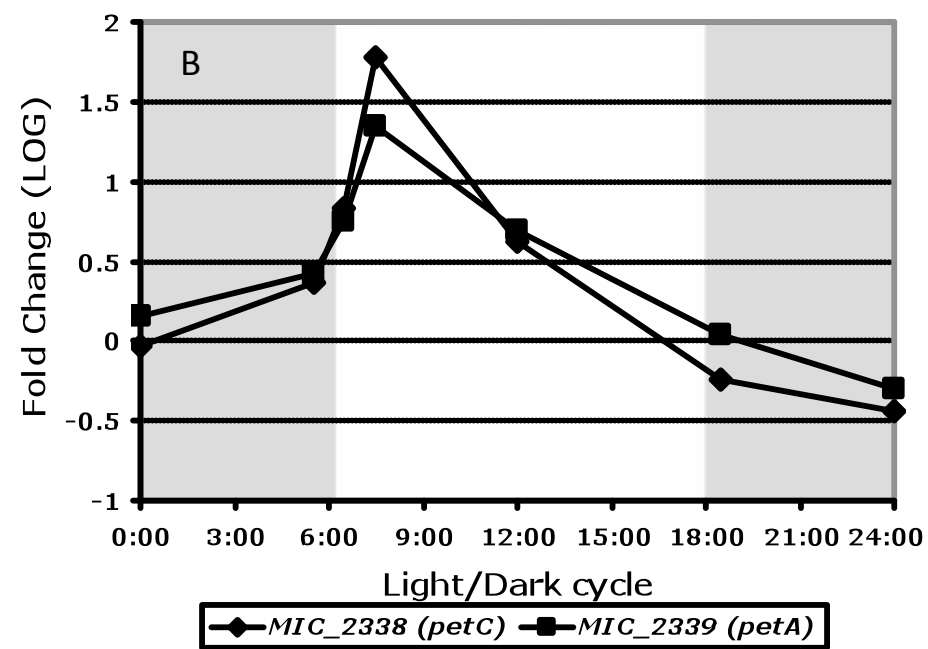

Supplement: Supporting Information S1 — 1. Design of our experiment. The figure shows the various sampling times during the light (yellow)/dark (gray) cycle. T1: 0:00; T2: 5:30; T3: 6:30; T4: 7:30; T5: 12:00; T6: 18:00; T7: 24:00. 2. Variations in the transcript abundance values obtained for one gene (hypD) during the light/dark cycle. For the six biological replicates (R1 to R6), all the fold change values obtained at each sampling time for the five oligonucleotide probes designed in this gene were plotted in the figure. The gray shaded part of the figure represents the dark period of the light/dark cycle. 3. Significant variations in the transcript abundance of genes encoding cytochrome b6f complex (pet genes) during the light/dark cycle. A: transcript abundance of the petBD gene cluster and B: transcript abundance of the petCA gene cluster. The gray shaded part of the figure represents the dark period of the light/dark cycle. (PDF) [file pone.0016208.s003.pdf]
